# Risk factors associated with sexually transmitted infections among HIV infected men who have sex with men

**DOI:** 10.1371/journal.pone.0170635

**Published:** 2017-02-03

**Authors:** Yun Xian, Bowen Zhu, Xuan Zhang, Ping Ma, Ye Wei, Hongli Xia, Wenjie Jiang, Changqing Yang, Xiaojun Meng, Peng Peng, Yue Yang, Liying Jiang, Minjie Chu, Xun Zhuang

**Affiliations:** 1 Department of Epidemiology and Biostatistics, School of Public Health, Nantong University, Nantong City, Jiangsu Province, China; 2 Wuxi Centre for Disease Control and Prevention, Wuxi City, Jiangsu Province, China; 3 Nantong Centre for Disease Control and Prevention, Nantong City, Jiangsu Province, China; 4 Department of Management Studies, University of Bath, Bath City, United Kingdom; 5 Yancheng Centre for Disease Control and Prevention, Yancheng City, Jiangsu Province, China; Fudan University, CHINA

## Abstract

To investigate the factors associated with sexually transmitted infection and Human Immunodeficiency Virus (STI-HIV) co-infection among men who have sex with men (MSM). A total of 357 HIV-infected participants (84 STI-HIV co-infection and 273 HIV infections only) were recruited from Jiangsu, China. Logistic regression analyses were used to estimate the related factors associated with STI-HIV co-infection. Marginal structural models were adopted to estimate the effect of transmission drug resistance (TDR) on STI-HIV co-infection. For all participants, logistic regression analyses revealed that those who diagnosed with HIV-1 for longer duration (≥1.8 years) were significantly associated with reduced STI-HIV co-infection risk (OR = 0.55, 95%CI: 0.32–0.96, *P* = 0.036). In further stratification analysis by antiretroviral therapy (ART), individuals with longer duration showed consistent significant associations with STI-HIV co-infection risk (OR = 0.46, 95%CI: 0.26–0.83, *P* = 0.010) among MSM with ART-naïve status. In addition, significant reduced risk for STI-HIV co-infection (OR = 0.98, 95%CI: 0.96–0.99, *P* = 0.010) were observed in younger (under the average age of 31.03) MSM of the same group. Interestingly, we also found TDR was significantly associated with an increased risk of STI-HIV co-infection risk (OR = 3.84, 95%CI: 1.05–14.03, *P* = 0.042) in ART-naïve group. Our study highlights a pattern of STI-HIV co-infection among MSM in China and indicates that targeted interventions aimed at encouraging TDR monitoring in MSM with early HIV infection are warranted.

## Introduction

Sexually Transmitted Infections and Human Immunodeficiency Virus (STI-HIV) co-infected individuals continue to expand at a rapid rate among men who have sex with men (MSM) in China. A previous review indicated that the syphilis prevalence in China among MSM increased from 6.8% (2003–2004) to 13.5% (2007–2008), whereas HIV prevalence rate increased from 1.3% (2003–2004) to 4.7% (2007–2008)[1]. STI also seems to contribute significantly to HIV burden inasmuch as it may facilitate HIV transmission by blocking protective mucosal barriers and recruiting susceptible immune cells (e.g, CD4+ T cells, macrophages) to the site of infection[2]. Similarly, HIV can accelerate progression of other STI and vice versa. When subjects are immune compromised, STI-HIV co-infection are more difficult to treat and disease periods may extend[3]. With the high incidence of syphilis among MSM (approximately 17 per 100 person years), and the heavy burden of STIs to HIV in China, it is important to figure out how these STI-HIV co-infection disease states impacted[4].

China was witnessing an increase in the proportion of MSM, especially they may play a bridge role in the spread of STI/HIV. In recent years, sexual exposure has become the primary route of HIV infection in China, and is the mode of transmission for STI infection[5]. Due to penile-anal intercourse and risk-related behavior, an approximately 45-fold higher risk of acquiring HIV was observed among MSM than other general male population in China[6]. STIs, associated with unsafe sex practices, intrinsic morbidity, have increased among MSM, while a recent research reported HIV-syphilis co-infection have increased from 1.4% during 2005–2006 to 2.7% during 2007–2008 among MSM in China[1]. Since STI and HIV share almost the same transmission routes and risk behaviors, strategies to control the epidemic of these infections should be combined together among MSM[7].

Currently, quite a few studies have aimed to monitor sexual behavior, STI-related knowledge, sexual network characteristics and health care consultation in HIV-infected MSM[8],[9],[10]. However, very few studies have explored molecular risk factors associated with STI-HIV co-infected MSM. Across the study, we analyzed the factors of molecular & socio-demographic risk factors among STI-HIV co-infected MSM, focusing on potential molecular risk factors. In addition, to overcome the time-varying covariates, we used marginal structural models to assessment the effect of risk factors on STI-HIV co-infected MSM. In studies, estimation of the causal effect of an exposure on an outcome may be biased because of confounding, i.e. covariates associated with treatment may also be associated with the potential response, so that the response differences cannot be attributed directly to the exposure. Proper estimation of causal effects must account for confounding. In studies where the exposure does not change, the traditional method of analysis is to model the probability of disease as a function of exposure. However, with a time-varying exposure, these traditional methods may be biased if time-varying covariates are simultaneously confounders and intermediates, if covariates are predictors of the outcome and also predict subsequent exposure, and past exposure history predicts resulting covariate level[11]. Such covariates are called time-dependent confounders, and they pose unique analytical challenges requiring specialized methods. To overcome the limitations of this standard approach, Robins and his colleagues[12]developed the marginal structural model. The advantage of MSM is that it can be adapted to unbiasedly estimate the causal effect of outcomes.

## Materials and methods

### Study population and blood sample

A total of 357 HIV-infected participants (84 STI-HIV co-infection and 273 HIV infection only controls) were recruited from 5 cities (Nantong, Suzhou, Taizhou, Wuxi and Yancheng) 2012 to 2015 in Jiangsu, China. HIV-infected individuals who had at least one of the STI were categorized into case group, while those without STI were categorized into control group. Demographic data including age, martial status, employment, and education status, HIV-1 subtype, CD4 at baseline and ART were directly collected from history cards of database of AIDS Direct Reporting Network System in the Center for Disease Control and Prevention (CDC) of 5 corresponding cities. Duration on HIV diagnosis was defined as the periods between the date of HIV diagnosis and the date of HIV-infected participants recruited, and categorized as two groups: ≤1.8 years and >1.8 years (the median of duration time). Marriage status was categorized as two groups: marital status and non-married status. CD4 count at baseline were categorized into three groups: < 500 cells/mm^3^, ≥ 500 cells/mm^3^ and unclear. The subtypes of HIV were categorized into three groups: CRF 01_AE, CRF 07_BC and others. The others were: CRF67_01B, CRF68_01B, CRF55_01B, CRF08_BC and subtype B. The information of STI were all confirmed by hospitals of county level and above after HIV diagnosis. Considering the influence of healthseeking behaviors, we further tested the association of the factors with STI-HIV co-infection risk. Two distinct subgroups were created: those taking ART and those without ART. Finally, blood samples of the 357 participants were obtained at HIV diagnosis, and plasma was isolated within 4 hours of sampling and stored at −80°C until HIV sequence genotyping.

### Viral RNA extraction, PCR amplification and sequencing

HIV-1 genome RNA was extracted from 200 μl of stored plasma specimens using the QIAmp Viral RNA Mini kit (Qiagen, Valencia, CA, USA) as Manufacturer’s instructions. Reverse transcription and nested polymerase chain (nPCR) amplification for partial genes of *pol* and *env* were performed by a home brew PCR procedure as described in our previous reports[13,14]. A one-tube reverse transcriptase polymerase chain reaction kit (GoldScript one-step RT-PCR kit, Life Technologies, USA), and PCR kit (TaKaRa Ex Taq Kit, Takara Biotechnology Co, Ltd; Dalian, China) were used according to the manufacture’s recommendations for amplification of the HIV-1 *pol* gene (protease 1–99 amino acids and part of reverse transcriptase 1–254 amino acids). The PCR amplification was carried out in a thermal cycler (GeneAmp PCR System 9700, Applied Biosystems, USA).

### Drug resistance

HIV-1 drug resistance mutations were determined according to the WHO 2009 Surveillance Drug Resistance Mutations (SDRM) list using the current Calibrated Population Resistance tool v5.0 of the Stanford University HIV Drug Resistance Database (http://cpr.stanford.edu/cpr.cgi)

### Statistical analyses

The χ^2^ test or fisher exact test for categorical variables were used to analyze distribution differences of demographic characteristics and selected variables between cases and controls. Odd ratio (OR) and their 95% confidence intervals (CI) were calculated by using logistic regression analyses to evaluate the association between STI-HIV co-infection and its related factors with an adjustment for age, marriage status, duration time, education, occupation and DR. To make results more interpretable, the marginal structural models were used to estimate the impact of TDR on STI-HIV co-infection.

All the statistics were performed by SPSS 16 software (IBM Company, New York, USA). Marginal Structural Models analyses were conducted using SAS software Version 9.3.

### Ethics approval and consent to participate

The study was reviewed and approved by the Institutional Review Board at the Human Medical Research Ethics Committee of the Center for Disease Control and Prevention in Nantong, Suzhou, Taizhou, Wuxi and Yancheng. All subjects were anonymous and provided written informed consent. The methods were carried out in accordance with the approved guidelines.

## Results

### Demographic characteristics of participants

A total of 357 participants were enrolled in the study, among which, 273 (76.47%) were infected with HIV only and 84 (23.53%) were STI-HIV co-infection. The distribution of selected characteristics between STI-HIV co-infection cases and controls among MSM were summarized in Table 1. Of these subjects, 92(25.77%) were on marital status, 265 (74.23%) were at non-married status. There were 102 (28.57%) participants had primary or junior school education, 116 (32.49%) were high school and 139 (38.94%) were post-graduate. HIV-infected individuals in our study were involved in different occupations in various proportions, in which students accounted for 5.60%. For the genotyping test, 214 (59.94%) infected with CRF01_AE, 80 (22.41%) infected with CRF07_BC, while other subtype (CRF67_01B, CRF68_01B, CRF55_01B, CRF08_BC, subtype B) were detected in 63 (17.65%) participants.

**Table 1:** Characteristics of 357 HIV infected MSM in Jiangsu.

Variable	Total sample, (n = 357), n (%)	STI-HIV co-infection, (n = 84), n (%)	HIV infection without STI, (n = 273), n (%)	χ^2^	*p*-value
**Age at diagnosis, years; Mean (SD)**	31.14±10.05	31.38±9.34	31.06±10.27	0.811	0.368
<30^+^	198 (55.46)	43 (51.19)	155 (56.78)		
≥30	159 (44.54)	41 (48.81)	118 (43.22)		
**Duration on diease**^++^**, years**				4.339	0.037
≤1.8	225 (63.03)	61 (94.05)	164 (60.07)		
>1.8	132 (36.97)	23 (5.95)	109 (39.93)		
**Marriage Status**				0.149	0.700
Non-married	265 (74.23)	61 (72.62)	204 (74.73)		
Married	92 (25.77)	23 (27.38)	69 (25.27)		
**Education**				0.081	0.960
Primary or junior school	102 (28.57)	23 (27.38)	79 (28.94)		
High school	116 (32.49)	27 (32.14)	89 (32.60)		
College or above	139 (38.94)	34 (40.48)	105 (38.46)		
**Occupation**				1.906	0.753
Students	20 (5.60)	4 (4.76)	16 (5.86)		
Workers and farmers	123 (34.45)	27 (32.14)	96 (35.16)		
Civil servants*	27 (7.56)	4 (4.76)	23 (8.42)		
Service industry**	104 (29.13)	26 (30.95)	78 (28.57)		
Others***	83 (23.25)	23 (27.38)	60 (21.98)		
**CD4 count, cells/mm**^**3**^				1.513	0.469
< 500	222 (62.18)	48 (57.14)	174 (63.74)		
≥ 500	123 (34.45)	32 (38.10)	91 (33.33)		
unclear	12 (3.37)	4 (4.76)	8 (2.93)		
**Region**				2.899	0.235
Southern Jiangsu^&^	250 (70.03)	65 (77.38)	185 (67.77)		
Middle Jiangsu^&&^	86 (24.09)	14 (16.67)	72 (26.37)		
Northern Jiangsu^&&&^	21 (5.88)	5 (5.95)	16 (5.86)		
**ART**				0.081	0.960
Yes	34 (9.52)	9 (10.71)	25 (9.16)		
No	323 (90.48)	75 (89.29)	248 (90.84)		
**Drug resistance**				3.025	0.105
Yes	14 (3.92)	6 (7.14)	8 (2.93)		
No	343 (96.08)	78 (92.86)	265 (97.07)		
**Subtype**				0.228	0.892
01_AE	214 (59.94)	50 (59.52)	164 (60.07)		
07_BC	80 (22.41)	18 (21.43)	62 (22.71)		
Others^#^	63 (17.65)	16 (19.05)	47 (17.22)		

^+^ The median of age;

^++^ Estimated as the period since the first seropositive HIV-1 antibody test date;

* Carders and staffs serving for governments, enterprises, school and hospital;

** People serving for commercial, catering and hair salon;

*** The unemployed, retirees and unclear;

^&^ Suzhou, Wuxi;

^&&^ Nantong, Taizhou;

^&&&^ Yancheng;

^#^ others refer to 67_01B, 68_01B, 55_01B, 08_BC, B

The mean age at diagnosis was 31.14 (±10.05) years old with a range of 18 to 67 years. DR was not significant with subtype of HIV-1 between two groups (*P* >0.05). Compared with controls, STI-HIV co-infected cases had a higher rate of short duration time (≤1.8 years) on diagnosis (94.05% vs 60.07%, *P* = 0.037). In addition, the education, occupation, CD count at baseline, region and ART between cases and controls had not statistic difference (P >0.05).

### Factors associated with STI-HIV co-infection

All of the variables between STI-HIV co-infection cases and controls were included in the logistic regression model (Table 2). For all participants, logistic regression analyses revealed that those who diagnosed with HIV-1 for longer course of disease (≥1.8 years) were significantly associated with STI-HIV co-infection risk (OR = 0.55, 95%CI: 0.32–0.96, *P* = 0.036 with adjustment for age, occupation, marriage status, education and DR).

**Table 2:** Logistic regression analyses of related factors associated with STI-HIV co-infection among MSM in Jiangsu.

Variable	STI-HIV co-infection rate, (N = 357), n (%)			STI-HIV co-infection rate on ART, (N = 34), n (%)			STI-HIV co-infection rate on ART-naïve (N = 323), n (%)		
	n/N (%)	OR (95% CI)	*p*-value	n/N (%)	OR (95% CI)	*p*-value	n/N (%)	OR (95% CI)	*p*-value
**Age at diagnosis, years; Mean (SD)**	31.14±10.05	0.99 (0.97–1.01)	0.263	32.18±10.77	0.91 (0.78–1.06)	0.231	31.03±9.98	**0.98 (0.96–0.99)**	**0.010**
<30^+^	43/198 (21.72)	ref		5/16 (31.25)	ref		38/182 (20.88)	ref	
≥30	41/159 (25.79)	1.28 (0.71–2.31)	0.412	4/18 (22.22)	1.06 (0.13–8.71)	0.956	37/141 (26.24)	1.34 (0.71–2.50)	0.365
**Duration on diease**^++^**, years**									
<1.8	61/225 (27.11)	ref		6/16 (37.50)	ref		55/209 (26.32)	ref	
≥1.8	23/132 (17.42)	**0.55 (0.32–0.96)**	**0.036**	3/18 (16.67)	0.28 (0.03–2.86)	0.283	20/114 (17.54)	**0.46 (0.26–0.83)**	**0.010**
**Marriage Status**									
Non-married	61/265 (23.02)	ref		7/24 (29.17)	ref		54/241 (22.41)	ref	
Married	23/92 (25.00)	1.20 (0.63–2.28)	0.588	2/10 (20.00)	1.25 (0.08–20.39)	0.874	21/82 (25.61)	1.40 (0.72–2.72)	0.325
**Education**									
Primary or junior school	23/102 (22.55)	0.84 (0.43–1.66)	0.621	1/12 (8.33)	0.10 (0.00–2.20)	0.144	22/90 (24.44)	0.99 (0.50–1.97)	0.054
High school	27/116 (23.28)	0.84 (0.45–1.56)	0.583	3/10 (30.00)	0.37 (0.03–4.62)	0.441	24/106 (22.64)	0.77 (0.41–1.44)	0.631
College or above	34/139 (23.46)	ref		5/12 (41.67)	ref		29/127 (22.83)	ref	
**Occupation**									
Workers and farmers	27/123 (21.95)	ref		3/12 (25.00)	ref		24/111 (21.62)	ref	
Students	4/20 (20.00)	0.70 (0.20–2.49)	0.581	0/2 (0.00)	NA	NA	4/18 (22.22)	0.52 (0.16–1.71)	0.281
Civil servants*	4/27 (14.81)	0.56 (0.17–1.86)	0.347	0/1 (0.00)	NA	NA	4/26 (15.38)	0.49 (0.15–1.59)	0.238
Service industry**	26/104 (25.00)	1.07 (0.61–1.89)	0.817	6/17 (35.29)	1.49 (0.13–16.66)	0.747	20/87 (22.99)	0.80 (0.46–1.37)	0.413
Others***	23/83 (27.71)	1.04 (0.38–2.91)	0.938	0/2 (0.00)	NA	NA	23/81 (28.40)	1.08 (0.38–3.09)	0.879
**Drug resistance**									
Yes	6/14 (42.86)	2.69 (0.86–8.40)	0.089	1/3 (33.33)	2.09 (0.09–47.03)	0.644	5/11 (45.45)	**3.84 (1.05–14.03)**	**0.042**
No	78/343 (22.74)	ref		8/31 (25.81)	ref		70/312 (22.44)	ref	

^+^ The median of age;

^++^ Estimated as the period since the first seropositive HIV-1 antibody test date;

* Carders and staffs serving for governments, enterprises, school and hospital;

** People serving for commercial, catering and hair salon;

*** The unemployed, retirees and unclear

In further stratification analysis by ART, individuals with longer duration showed consistent significant associations with STI-HIV co-infection risk (OR = 0.46, 95%CI: 0.26–0.83, *P* = 0.010 with adjustment for age, occupation, marriage status, education and TDR) among MSM with ART-naïve status. In addition, significant reduced risk for STI-HIV co-infection (OR = 0.98, 95%CI: 0.96–0.99, P = 0.010 with adjustment for duration time on diagnosis, occupation, education, marriage status, and TDR) were observed in young (under the average age of 31.03) MSM of the same group. Interestingly, we also found TDR was significantly associated with an increased risk of STI-HIV co-infection risk (OR = 3.84, 95%CI: 1.05–14.03, *P* = 0.042 with adjustment for age, duration time on diagnosis, marriage status, education, occupation) of this group. However, no obvious variable was associated with STI-HIV co-infection among antiretroviral experienced MSM (Table 2).

### Distribution of antiretroviral drug resistance

**Fig 1:**
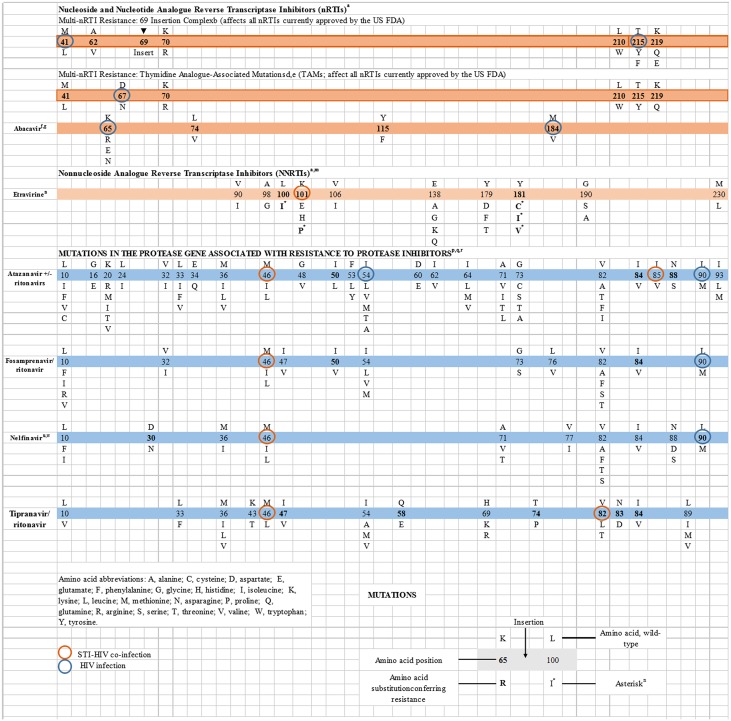
Drug resistance mutation among HIV-infected MSM in Jiangsu.

DR were detected in 14 of 357 subjects (3.9%). Among the 34 ART-receipt individuals, 3(8.8%) had evidence of resistance to at least one antiretroviral drug. For HIV-infected participants of ART-naïve status, 11(3.4%) with transmitted drug resistance were found. Resistance was observed most frequently for the PI (atazanavir +/- ritonavirs, fosamprenavir/ ritonavir, nelfinavir, tipranavir/ ritonavir). (Fig 1). We observed STI-HIV co-infection more frequently in participants with TDR (6.7%) and M46L was detected at higher frequency (40.0%) than any other mutation in STI-HIV co-infected participants.

### Estimation of Marginal Structural Models

**Fig 2:**
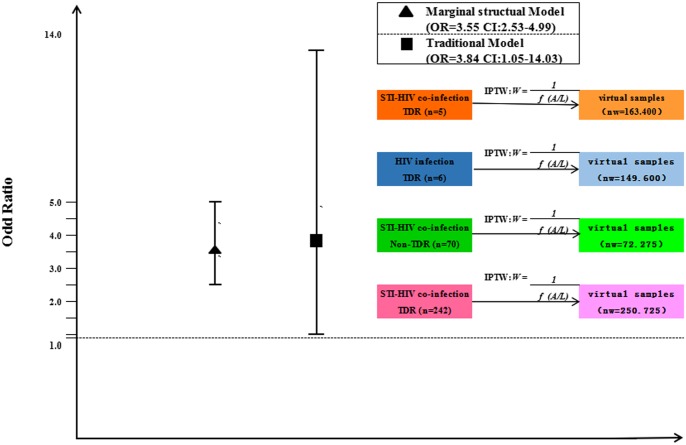
Odd ratio from both traditional model and marginal structural model (MSM) for the risk of STI-HIV co-infection.

Applying Marginal Structural Models, TDR was estimated to increase frequency in STI-HIV co-infection (OR = 3.55, 95%CI: 2.53–4.99, *P*<0.001). Though it was slightly lower than estimate from traditional models (OR = 3.84, 95%CI: 1.05–14.03, *P* = 0.042), the marginal structural models significantly increased the statistical power of this study (Fig 2).

## Discussion

In our research group, the prevalence rate of STI among HIV-infected MSM was 23.52% (84/357), this burden of STI is higher than Asia (median 17.4%) and Europe (median 14.7%), and greater than a meta-analysis (median 16.3%) in all HIV population in China[2]. The difference may result from different target population, since all the subjects in our study were MSM, who were regarded as high-risk behavior group of STI infection especially for anal intercourse (AI). One study showed that AI may give rise to STI (OR = 2.42). Additional, when compared with population of Asian, Europe and China, the sample size of our studied might not have been large enough to achieve a convincing conclusion about the prevalence of STI among HIV-infected MSM.

The logistic analysis found that subjects with longer duration had a significant decreased risk for STI-HIV co-infection risk with a total of 84 cases and 273 controls. After stratification by ART, two variables of age at diagnosis and duration time on diagnosis showed a significant association with STI-HIV co-infection, and TDR was associated with a significant increased STI-HIV co-infection risk in a case-control study of 75 STI-HIV co-infection cases and 248 HIV infection controls among MSM with ART-naïve status. In contrast, there is no significant association with STI-HIV co-infection risk among antiretroviral experienced MSM. To our knowledge, this is the first association study of TDR and STI-HIV co-infection risk among MSM.

In our research, we found a significant association of duration time with STI-HIV co-infection risk, which indicated MSM with early HIV infection may have potential threats of STI/HIV transmission to their partners, thus it is necessary that HIV care providers may be more pro-active in assessing ongoing risk behavior and suggesting STI testing among their clients. In addition, a recent research indicated that defective proviruses accumulate rapidly within the early period of infection to make up over 93% of all proviruses, hence promote harmful immune activation[15]. It is plausible that the weak individual immune system could further lead STI in HIV infected individuals, which may partly explain our result.

In addition, we also found young HIV infected MSM was associated with a significant decreased STI-HIV co-infection risk. There are several possible reasons for the results in our study. First, the young HIV-1 infected MSM were more acceptable to take protective measures once they had some information that suggested its potential as a useful intervention. Second, when compared with older population, the immune systems of younger population were stronger. The different level of immune system may be the potential reason for different ssusceptibility to STI among HIV-infected MSM.

Interestingly, our study showed STI-HIV co-infection occurred more frequently in individuals with TDR population. Previous studies showed a significant correlation between DR mutations and overlapping polymorphisms in the reverse transcriptase (RT) and protease (PR) viral genes[16], which can partly explain our result, high variation of HIV virus or rebuilt the HIV-specific CD4 or CD8 T-cell damage immune response for HIV infected individuals[17]. Key research priorities recently identified suppression of viral replication can reduce harmful immune activation, slowing pathogenesis, and stem the inhibition of CD4+T cells (‘treatment-as-prevention’) CD4 + T cells generated IL—2 and IFN–γ, enhancing the individual immune system, which further protect HIV infected individuals from STI. In particular, up to date, no direct experimental evidence has been reported for the hypothesis and future studies are warranted to prove the point[18],[19]

The prevalence of TDR among MSM in our study was 3.4% (11/323), which was lower than that of worldwide (10 ~ 20% of HIV-1 infection worldwide)[20]. According to the present investigation, the occurrence of drug resistance mutations both in viruses from individuals receiving ART and in transmitted viruses to ART-naïve individuals is increasing at a global level[21],[22] It is noteworthy that the prevalence of TDR is low in resource-limited settings, however it maybe show an increasing trend in recent years. That would result a heavy disease burden of STI to HIV infection as access to ART has been expanding[23]. Considering the dramatic health threats by sexually transmitted co-infections posing to people living with HIV/AIDS in recent years, the current study suggested surveillance drug resistance mutations (SDRMs) as a national surveillance system which was largely consistent with our suggestion[24]. Other studies further emphasize the association between TDR and HIV subtype among HIV infected MSM, which may be meaningful for STI control[25],[26],[27],[28].

It is possible that the results of our study could be affected by time-depending bias given that some of the participants were of a long duration time on disease and hence could not afford TDR or not which consequently switch to STI-HIV co-infection. Therefore, we used marginal structural models to estimate the causal effect of TDR on STI-HIV co-infection[29]. For MSM participants, TDR was estimated to increase the possibility to infect with STI in HIV by 3.552. This was slightly lower than the estimates from traditional association model. The results indicated that TDR status play an exceedingly promotive influence on STI-HIV co-infection after controlling time confounder.

Our study results should be interpreted in the context of some limitation. First, STIs can present in individuals without causing symptoms, so-called asymptomatic infections, and still potentially be transmitted to others, resulting in selection bias[30]. Second, the number of HIV infected MSM on ART status in our study was limited, leading to a difficult to further draw conclusion about acquired drug resistance (ADR) associated with STI-HIV co-infection. Studies with larger sample sizes are needed to evaluate our findings.

Despite the limitation above, our research also had several advantages. First, we shift the attention from sexual behaviors such as condom use and number of sex partners to molecular level of HIV for STI-HIV co-infection. Furthermore, the supplementary data analyse (marginal structural models) to control for confounding improved the robustness of the finding. When exposure level in a variable included in the research reaches a certain value, leading to all samples under a fixed exposure levels, Inverse Probability Of Treatment Weighting (IPTW) would produce deviation. It makes the strong assumption of no unmeasured confounders[31]. The marginal structural models can correctly adjust for measured time-varying confounders that were affected by exposure.

## Conclusions

In summary, this research indicates the role of TDR in STI-HIV co-infection risk in HIV infected MSM as well as age and duration time with STI-HIV co-infection risk. Our study highlights a pattern of STI-HIV co-infection among MSM in Jiangsu and indicates that targeted interventions aimed at encouraging TDR monitoring in MSM with early HIV infection are urgently needed. Moreover, further researches should focus on TDR mechanisms on the etiology of STI-HIV co-infection development and illuminate TDR monitoring can be targeted for prevention strategies to mitigate the obviously increased prevalence of STI-HIV co-infection among MSM.

## Supporting information

S1 File Original data. (XLS)

## Abbreviations

ART: antiretroviral therapy
DR: Drug resistance
IPTW: Inverse Probability Of Treatment Weighting
MSM: Men who have sex with men
PR: protease
RT: reverse transcriptase
SDRM: Surveillance drug resistance mutations
STI: Sexually transmitted infection
TDR: Transmission drug resistance
