# Correction: Risk factors associated with sexually transmitted infections among HIV infected men who have sex with men

**DOI:** 10.1371/journal.pone.0176579

**Published:** 2017-04-20

**Authors:** The PLOS ONE Staff

The following information is missing from the Funding section: This work was funded by the National Natural Science Foundation of China (81373060), the Six Major Human Resources Project of Jiangsu Province (WSN-015), Preventive Medicine Project of Jiangsu Province (Y2013071) and the Science and Technology Program of Nantong City (MS12015125). The publisher apologizes for the error.
